# Language Proficiency and Migrant–Native Disparities in Postpartum Depressive Symptoms

**DOI:** 10.3390/ijerph18094782

**Published:** 2021-04-29

**Authors:** Sousan Hamwi, Elsa Lorthe, Henrique Barros

**Affiliations:** 1 EPIUnit–Instituto de Saúde Pública, Universidade do Porto, 4050-091 Porto, Portugal; henrique.barros@ispup.up.pt; 2 Unit of Population Epidemiology, Department of Primary Care, Geneva University Hospitals, 1205 Geneva, Switzerland; elsa.lorthe@gmail.com; 3 Epidemiology and Statistics Research Center/CRESS, INSERM, INRA, Université de Paris, F-75004 Paris, France; 4 Departamento de Ciências de Saúde Pública e Forenses e Educação Médica, Faculdade de Medicina da Universidade do Porto, 4200-450 Porto, Portugal

**Keywords:** postpartum depression, mental health, migrant, pregnancy, communication barriers, language proficiency, health equity

## Abstract

Migrant women have a higher risk of developing postpartum depressive symptoms (PPDS) than do native women. This study aimed to investigate the role of host-country language proficiency in this disparity. We analysed the data of 1475 migrant and 1415 native women who gave birth at a Portuguese public hospital between 2017 and 2019 and were participants in the baMBINO cohort study. Migrants’ language proficiency was self-rated and comprised understanding, speaking, reading, and writing skills. PPDS were assessed using the Edinburgh Postnatal Depression Scale with a cut-off score of ≥10. Multivariable logistic regression models were fitted to estimate the association between language proficiency and PPDS. PPDS were experienced by 7.2% of native women and 12.4% among migrants (*p* < 0.001). Increasing proportions of PPDS were observed among decreasing Portuguese proficiency levels; 11% among full, 13% among intermediate, and 18% among limited proficiency women (*p*trend < 0.001). Full (aOR 1.63 (95% CI 1.21–2.19)), intermediate (aOR 1.68 (95% CI 1.16–2.42)), and limited (aOR 2.55 (95% CI 1.64–3.99)) language proficiencies were associated with increasingly higher odds of PPDS among migrant women, compared to native proficiency. Prevention measures should target migrant women at high risk of PPDS, namely those with limited language skills, and promote awareness, early detection, and help-seeking, in addition to facilitating communication in their perinatal healthcare encounters.

## 1. Introduction

Postpartum depression (PPD) is a non-psychotic depression that affects about 17% of healthy mothers within one year of childbirth [1,2,3]. The exact aetiology of PPD is still unknown, but literature suggests a combination of genetic, physical, psychosocial, and obstetric risk factors [4,5]. If left undetected or untreated, PPD can have serious adverse consequences on women’s health, on the maternal–infant bond, on the child’s cognitive and emotional development, and on the family well-being as a whole [6,7,8,9].

It is well established that migrant women are more likely to suffer from PPD than are native women [10,11,12,13]. A meta-analysis showed that the risk of experiencing postpartum depressive symptoms (PDDS) is 1.5–2 times higher among migrant women than among natives [14]. Reasons behind this difference are likely to be of a complex nature, including unfamiliarity with the host-country and migration-related stress, poor marital adjustment or abuse, low socioeconomic status, lack of social support, and reduced access to health and social services [14,15].

While the migrant population is globally increasing and diversifying, language barriers are more frequently encountered in clinical practice and are gaining more attention in public health research [16]. Nevertheless, despite being one of the most documented barriers to maternal healthcare access and utilisation among migrants, literature providing a comprehensive assessment of language barriers’ effect on migrants’ perinatal health is scarce [16], and no previous study has investigated the association between host-country language proficiency and PPDS. The reason could be that women who do not speak the host-country language are often excluded from studies [17], as their inclusion could be costly and challenging and research tools may not be validated or available in many languages [18].

An additional limitation lies in the heterogeneity of language skills’ measurement in the existing literature. Many studies define local language competence simply according to the preference to conduct the study interview in that language [19,20]. On the other hand, many studies from the United States refer to Limited English Proficiency as speaking English “less than very well”, based on the classification of the United States Census Bureau [21,22]. This means that migrants of intermediate, limited, and no language proficiency are all considered non-proficient, which is an oversimplification of the reality and might dilute the language barrier effect, especially for the least proficient migrants.

Studying migrant and native women in Portugal, we aimed to answer the following question: Does host-country language proficiency influence the rate of PPDS?

## 2. Materials and Methods

### 2.1. Study Design

This study was based on data collected within the scope of baMBINO (Perinatal Health in Migrants: Barriers, Incentives, and Outcomes)—A Portuguese prospective cohort study. baMBINO aimed to investigate the maternity experiences and outcomes of migrant women, defined as foreign-born women, compared to those of native women. A more detailed description of baMBINO is provided elsewhere [23].

### 2.2. Setting

In Portugal, the universal healthcare system enables all women to have free access to health services, including migrants, regardless of their legal status. Except for emergency health situations, women in their postpartum period should seek healthcare through their primary healthcare centres and family doctors [24]. Speciality care in the National Health Service, like mental health services, is based on referrals from women’s family doctors or any other specialist. There are orientations but no specific national guidelines relating to the prevention or management of PPD in Portugal [24]. It is also worth noting that professional interpretation services are not often available in a timely manner in the healthcare settings in Portugal, and either ad-hoc interpreters like patients’ family members or friends, automated translation services like Google Translate, or shared languages like English are commonly used to facilitate communication with non-Portuguese speakers.

### 2.3. Participants

Eligibility criteria in baMBINO included adult migrant and native women who had a live birth in one of the 32 (out of 39) collaborating Portuguese maternity units between April 2017 and March 2019.

Overall, 5431 women consented to participate in baMBINO, out of which 2863 were migrants. Contact information and clinical data of enrolled women were collected from their medical records. A team of trained multi-lingual interviewers later contacted those women to complete a computer-assisted telephone interview (median months from delivery to interview, 2; interquartile range, 3 to 4). Women were interviewed in their preferred language, using interpreters when needed, and over 90% of the interviews took place within six months of recruitment. The Edinburgh Postnatal Depression Scale (EPDS) [25] was administered during this interview, along with the Migrant-Friendly Maternity Care Questionnaire (MFMCQ) [26], covering maternity care and migration experience topics, including proficiency in the Portuguese language.

### 2.4. Study Population

**Figure 1:**
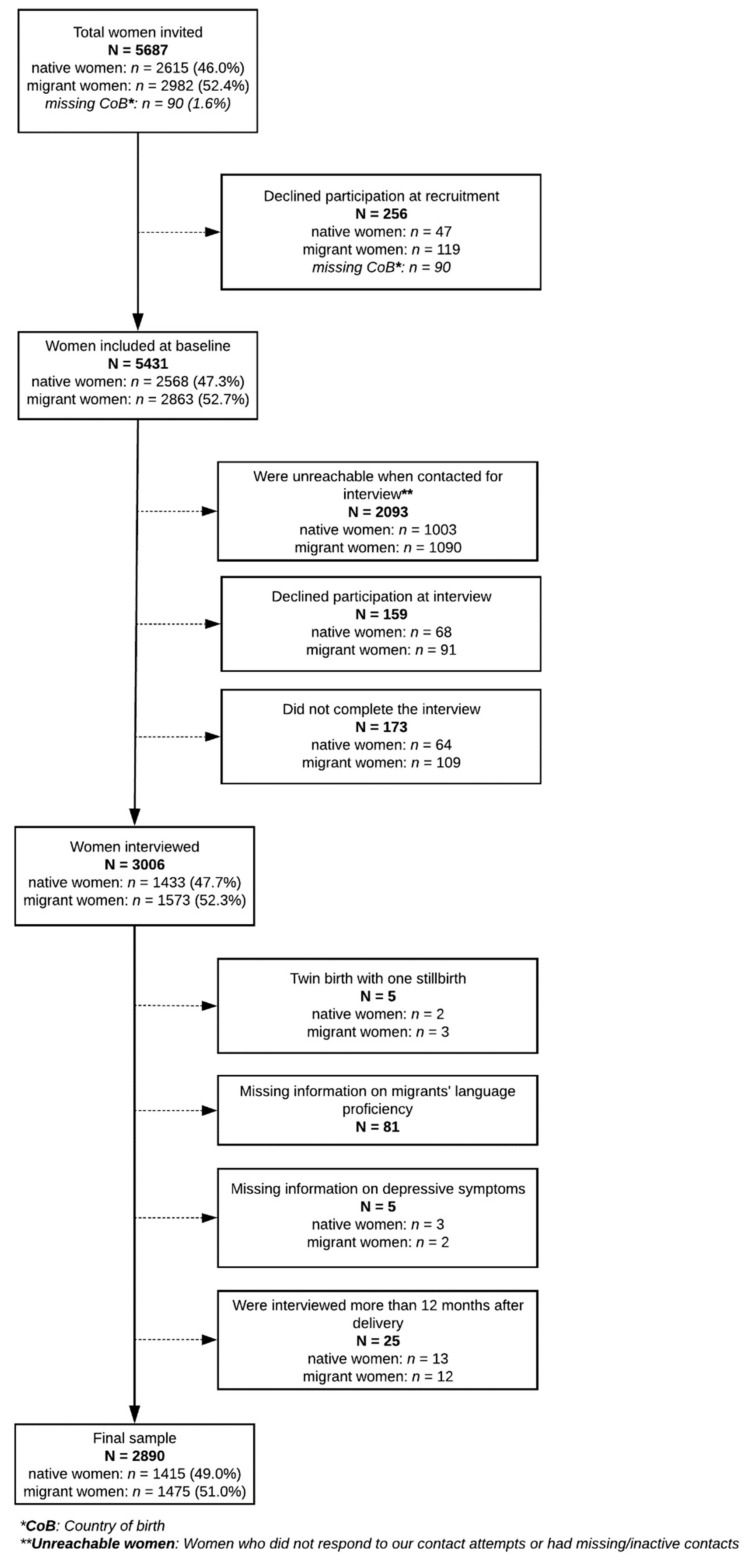
Flowchart of participation throughout the baMBINO study, Portugal.

For this study, all interviewed baMBINO participants were considered eligible (*n* = 3006). We excluded women who had a twin birth with one stillbirth (*n* = 5), did not report their Portuguese proficiency level (*n* = 81), did not respond to the EPDS (*n* = 5), or were interviewed more than 12 months after birth (*n* = 25) (Figure 1).

### 2.5. Exposure Measure

The host-country language (Portuguese) proficiency was our exposure of interest and was categorised into native, full, intermediate, or limited. Portuguese-born women were assumed to have native proficiency. In contrast, migrant women were asked, as part of the MFMCQ, to rate four components of their proficiency (understanding, speaking, reading, and writing) based on the following scale: 0 (no proficiency), 1 (limited proficiency), 2 (intermediate proficiency), and 3 (full proficiency). Category 0 contained a small number of women (*n* = 25 in understanding, *n* = 39 in speaking, *n* = 43 in reading, and *n* = 57 in writing), so we combined it with category 1. Finally, the mode of the four components’ values for each woman was defined to be her final overall proficiency score. We assumed that women had an intermediate proficiency when the set of values was bimodal (*n* = 77). We also assumed full proficiency for Brazil-born women where Portuguese is the primary spoken language, but not for women born in Portuguese speaking African countries due to the wide range of local spoken languages there, in contrast to Brazil. Oral proficiency scores (including only speaking and understanding) were consistent with the overall proficiency scores, so we only used the overall scores in our analysis.

### 2.6. Outcome Measure

Our primary outcome was self-reported postpartum depressive symptoms (PPDS), assessed using the Edinburgh Postnatal Depression Scale (EPDS)—A ten-item questionnaire that reflects a woman’s mood in the week before administration with a final score ranging from 0 to 30 [25]. The cut-off score we used as an indicator of PPDS was ≥10.

### 2.7. Covariates

The following covariates were considered in the analyses: women’s migration-related characteristics (migrant status (foreign-born vs. native-born), the region of birth according to the World Bank classification, length of stay in Portugal, and legal status in Portugal), economic and sociodemographic characteristics (maternal age, marital status, highest educational level attained, monthly household income per person, and having an individual health insurance plan apart from access to the National Health Services, which is available to all women (no vs. yes)), obstetric characteristics (parity, previous pregnancy complications, smoking during the index pregnancy, complications during the index pregnancy, twin pregnancy, delivery with an obstetric intervention (instrumented delivery (vacuum or forceps), episiotomy, or caesarean section), adverse neonatal outcomes (preterm birth (<37 gestational weeks), low birth weight (<2500 g), congenital malformation, or admission to a neonatal intensive care unit), and time elapsed since delivery).

### 2.8. Statistical Analysis

We first compared the characteristics of women by their interview status. We then described the frequencies and proportions of participants’ characteristics and compared them across language proficiency and depression risk groups (EPDS scores 0–9 vs. ≥10) using the chi-square or Fisher exact tests, as appropriate. We tested for a trend of the effect of language proficiency on PPDS in the study population. We also compared the mean values of the log-transformed EPDS scores across language proficiency groups using the one-way analysis of variance (ANOVA).

We investigated the association between language proficiency and an EPDS score ≥10 using univariate and multivariable logistic regression models and reported the results as crude (OR) and adjusted odds ratios (aOR) with their 95% confidence intervals (CI).

We chose the covariates included in the final multivariable models based on their relevance according to the literature and the best statistical fit for the models. Covariates included maternal age, highest educational degree attained, marital status, having an individual health insurance plan, parity, delivery with an obstetric intervention, and adverse neonatal outcomes.

Among the variables included in the models, the percentage of missing data ranged from 0 to 4.4%. We conducted our analyses on complete-cases and then applied multiple imputations by chained equations (MICE) [27]. The imputation model involved using the exposure, the outcome, and all covariates of the multivariable models. Associations were estimated within each of the 50 imputed data sets generated with 20 iterations, and results were pooled in a single estimate according to Rubin rules.

We performed a sensitivity analysis by restricting our population to natives and recent migrants—those living in Portugal for ≤5 years—to disentangle the effect of language proficiency on PPDS from the possible effect of the improved acculturation and social support due to long durations of stay in the host country. We conducted additional sensitivity analyses using the EPDS cut-off values ≥11 and ≥13 to verify the robustness of our results.

All data were analysed using Stata 15.0 (StataCorp LP, College Station, TX, USA). Statistical significance was set at two-tailed *p* < 0.05.

## 3. Results

Among the 5431 women enrolled in baMBINO, 3006 (55%) were interviewed and considered eligible for this study, and 2890 (96%) were included in the final analysis (Figure 1).

There were significant differences in most maternal characteristics by their interview status. Women who were not interviewed were most often from Portuguese-speaking African countries, young (18–24 years old), single, of low education (<12th grade), multiparous, smokers during pregnancy, and with no interventions during delivery. On the other hand, there were no significant differences in terms of their migrant status (being native or migrant), having twin pregnancy, complications during pregnancy, or adverse neonatal outcomes (Table S1).

Among women included, 1475 (51%) were migrants, out of which 874 (59.3%) had full proficiency in Portuguese, 412 (27.9%) had intermediate proficiency, and 189 (12.8%) had limited proficiency (Table 1).

The proportions of women having PPDS (EPDS scores ≥ 10) were overall greater among migrants than among native women (7.2% vs. 12.4%, *p* < 0.001), with increasing proportions of PPDS with decreasing language proficiency levels. Among native women, 7.1% had EPDS scores ≥10, compared to 11.3%, 12.6%, and 18.0% among migrants with full, intermediate, and limited Portuguese proficiency levels, respectively (*p*trend < 0.001) (Table 2).

We also found increasing means of log-transformed EPDS scores with decreasing language proficiency (*p*trend < 0.001), with the lowest value being for native women and the highest for migrants with limited Portuguese competence (1.02, 1.19, 1.21, and 1.29 for native, full, intermediate, and limited language skills, respectively).

Women’s language proficiency varied according to most of the maternal characteristics studied: region of birth, length of stay in Portugal, legal status, age, marital status, education level, income, health insurance, obstetric history, smoking during pregnancy, delivery with any obstetric intervention, and time elapsed since delivery (Table 1). On the other hand, the maternal characteristics associated with an EPDS score of ≥10 were: being Asian- or European-born (excluding Portugal), a recent arrival to Portugal (≤5 years), low monthly household income (<500 EUR/person), non-users of individual health insurance plans, and no obstetric interventions during recent birth (Table 1).

No significant differences in PPDS were found according to age, marital status, legal status, education level, obstetric history, smoking or complications during pregnancy, twin pregnancies, adverse neonatal outcomes, or time elapsed since delivery (Table 1).

Analyses after multiple imputation produced similar results to those of complete-case analyses. Full, intermediate, and limited Portuguese skills were associated with higher risks of PPDS (EPDS scores ≥10); aOR 1.63 (95% CI 1.21–2.19); aOR 1.68 (95% CI 1.16–2.42); aOR 2.55 (95% CI 1.64–3.99) compared to native Portuguese, respectively (Table 2).

When restricting the analysis to recent migrants, the effect of language proficiency on PPD risk was generally stronger than when considering the overall migrant group (Table 2). Similar results were found when using the cut-offs 11 or higher and 13 and higher (Table S2).

## 4. Discussion

Our study confirms a higher risk of postpartum depressive symptoms among migrant women in Portugal than among native women. Considering relevant socioeconomic and obstetric variables, migrants’ risk of having PPDS increases as their host-country language proficiency decreases, particularly among newcomers. These findings were robust to the different EPDS cut-off values used to define PPDS.

Similarly to what we observed, an Australian study that compared the postpartum experiences of foreign-born to native-born women found that migrants who had less than proficient English skills were 2.4 times more likely to have an EPDS score ≥13 than did native speakers [28]. In another Canadian study, migrant women who could not speak English or French had double the PPDS risk (EPDS score ≥10) of those who could speak either [29].

A systematic review reported different findings on the association between acculturation and PPDS among primarily Hispanic migrants in the United States [19]. Most studies included in the review showed no significant association between language preference for the interview and PPDS in the adjusted models [19]. However, these studies had small sample sizes (ranging from 66 to 377), mixed native-born with foreign-born Hispanic women in the samples, and used the interview language preference, which is not always a good measure of host-country language skills, considering that even proficient women could prefer to be interviewed in their native tongue. Likewise, another US study on migrant mothers of Arabic descent showed no effect of language preference on PPDS [30].

We propose different hypotheses through which language barriers can contribute to PPDS risk disparities.

At the individual level, the decreased ability to communicate using the local language might be associated with a higher sense of isolation, stigma, dependence on others to assist with daily activities, and a lack of social support, which is usually much needed in the postpartum period, thus leading to the development of depressive symptoms [28,31]. This could be especially true for recent and minority migrant groups, as they will probably have fewer means of social support than those who have been in the country for a long time and are more acculturated and more familiar with the health system, which is supported by our results.

At the healthcare level, having to use a second language in the healthcare settings is shown to be associated with language-specific health communication anxiety, and this anxiety is accompanied by a reluctance to seek out health services [32]. This effect was found to be significantly stronger for mental health than for physical health contexts [32]. In addition, limited local language ability, which can add up to limited comprehension of medical terminologies, has been shown to be associated with feelings of discrimination and reduced mental health services access and use [33,34,35,36,37,38]. These are some of the indirect pathways that lack of language proficiency could be contributing to increasing the burden of undetected, untreated PPDS among migrants. Additionally, language proficiency is central to establishing a rapport between patients and mental healthcare providers and unravelling something as subtle, complicated, and intimate as emotions, which is necessary to achieve proper diagnosis and treatment [39].

### 4.1. Strengths and Limitations

We believe that a main strength of this study is that it links up perspectives and concepts of various disciplines, namely linguistics/psycholinguistics and psychology/psychiatry.

The interviews were conducted in the women’s language of preference, which allowed the inclusion of non-proficient migrant women. Migrants’ self-rated language proficiency measurement in our study was more comprehensive than most of the literature that simply categorises women as being a speaker or a non-speaker of the host-country language. Our language proficiency measure combined different components and proficiency levels, which could better reflect the heterogeneity of migrants’ linguistic competence and the potentially consequent PPDS disparities that might not have been detected otherwise.

Moreover, to assess PPDS, we applied the most widely validated and used depression screening tool among clinicians and researchers in perinatal care: the Edinburgh Postnatal Depression Scale. The EPDS was administered using the validated versions compatible with the women’s languages. We also chose the EPDS cut-off value recommended in the original validation study [25] and across the different cultures and languages of women in our study population (≥10). Additionally, we tested the other recommended EPDS cut-off values of ≥11 (maximising combined sensitivity and specificity) and ≥13 (maximising specificity), based on a recently published meta-analysis of individual participant data [40], which attests to the robustness of our results.

A limitation of this study is that, due to a high attrition rate, many characteristics of the interviewed women differed significantly from those not interviewed. This could possibly restrict the external validity of our study results.

While language skills were not measured objectively, we believe that perceived communication barriers are more relevant in the scope of PPD and can also be utilised as a simple screening measure to identify migrant women at high risk of PDDS.

We had no information on whether women had strong social support or sought mental health services. This could have, consequently, attenuated the association between limited language skills and PPDS in our study. Showing stronger associations across recent migrants, who are less likely than long-term migrants to have a strong social support network, supports this conclusion.

Finally, we had no information on whether women could communicate with their community or healthcare providers in other shared languages such as English, in which case the effect of language barriers would have also been attenuated.

### 4.2. Implications for Health Policy and Future Research

To live up to the United Nations Member States’ 2020 pledge to “Leave No One Behind” [41]—a commitment to prioritise and accelerate the progress of those furthest behind—it is vital to design and implement migrant-targeted health policies that aim to address both migrant–native and migrant–migrant maternal health inequities. Identifying and characterising the different PPD risk groups within migrant women could be an essential step in approaching these equities.

Public health prevention measures should aim to identify and reach migrant women at high risk of PPD during pregnancy and after birth—particularly those who arrived recently to the host country or have limited language skills—and promote awareness, early identification of PPDS, and seeking mental health services if needed. This would serve to decrease the burden of undetected and untreated PPDS in these groups and, consequently, the possible long-term adverse consequences.

Additionally, health policies aiming to facilitate migrant women’s communication with their healthcare providers and addressing language barriers experienced during the pregnancy and postpartum periods could be essential steps in reducing PPDS risk among vulnerable migrant women groups and approaching equity in maternal mental health between migrant and native women.

Future research to better understand the mechanisms linking language proficiency to PPDS are needed. For example, it will be interesting to investigate how the quality of communication influences the association between language proficiency and PPDS.

## 5. Conclusions

Host-country language proficiency plays a role in PPDS migrant–native disparities even when considering socioeconomic and obstetric variables. Public health measures should aim to identify and reach migrant women at high risk of PPDS, namely recent migrants and those with limited language skills, and promote awareness, early identification of PPDS, and seeking mental health services if needed. These measures, along with efforts to facilitate women’s communication with healthcare providers, would constitute essential milestones in the journey of achieving maternal health equity.

## Figures and Tables

**Table 1:** Maternal characteristics by host-country language proficiency and EPDS score (*n* = 2890).

Characteristics	Language Proficiency				*p*	EPDS Score		*p*
	Native (*n* = 1415)	Full (*n* = 874)	Intermediate (*n* = 412)	Limited (*n* = 189)		0–9 (*n* = 2604)	≥10 (*n* = 286)	
	*n* (%)	*n* (%)	*n* (%)	*n* (%)		*n* (%)	*n* (%)	
Migration-related characteristics								
Region of birth (*n* = 1475)								
Brazil	-	325 (37.2)	0 (0.0)	0 (0.0)		284 (22.0)	41 (22.2)	
PALOP ^a^	-	357 (40.9)	256 (62.2)	45 (23.8)		588 (45.6)	70 (37.8)	
Other African countries	-	6 (0.7)	15 (3.6)	19 (10.1)	<0.001	33 (2.6)	7 (3.8)	<0.001
Europe	-	154 (17.6)	106 (25.7)	58 (30.7)		274 (21.2)	44 (23.8)	
Asia	-	2 (0.2)	13 (3.2)	56 (29.6)		50 (3.9)	21 (11.3)	
America	-	30 (3.4)	22 (5.3)	11 (5.8)		61 (4.7)	2 (1.1)	
Length of stay in Portugal (*n* = 1466)								
≤5 years	-	254 (29.2)	164 (40.2)	146 (77.2)		474 (36.9)	90 (49.2)	
5–10 years	-	192 (22.1)	135 (33.1)	34 (18.0)	<0.001	321 (25.0)	40 (21.8)	0.005
>10 years	-	423 (48.7)	109 (26.7)	9 (4.8)		488 (38.1)	53 (29.0)	
Migrant legal status (*n* = 1468)								
Citizenship	-	381 (43.8)	128 (31.1)	14 (7.4)		466 (36.3)	57 (31.0)	
Permanent residency/EU citizen	-	130 (15.0)	79 (19.2)	33 (17.6)	<0.001	213 (16.6)	29 (15.8)	0.32
Temporary residency	-	293 (33.7)	175 (42.6)	114 (61.6)		504 (39.2)	78 (42.4)	
Undocumented migrants	-	65 (7.5)	29 (7.1)	27 (14.4)		101 (7.9)	20 (10.9)	
Sociodemographic characteristics								
Age (years) (*n* = 2890)								
18–24	182 (12.9)	140 (16.0)	74 (18.0)	37 (19.6)		384 (14.7)	49 (17.1)	
25–34	780 (55.1)	473 (54.1)	239 (58.0)	117 (61.9)	<0.001	1452 (55.8)	157 (54.9)	0.54
≥35	453 (32.0)	261 (29.9)	99 (24.0)	35 (18.5)		768 (29.5)	80 (28.0)	
Marital status (no partner) (*n* = 2890)	341 (24.1)	234 (26.8)	148 (35.9)	21 (11.1)	<0.001	665 (25.5)	79 (27.6)	0.44
Highest education level attained (*n* = 2889)								
Post-secondary (>12th grade)	567 (40.1)	285 (32.6)	123 (29.9)	80 (42.6)		951 (36.5)	104 (36.4)	
Upper secondary (12th grade)	482 (34.0)	399 (45.7)	122 (29.6)	38 (20.2)	<0.001	953 (36.6)	88 (30.8)	0.10
Lower secondary (9th grade)	297 (21.0)	146 (16.7)	110 (26.7)	32 (17.0)		518 (19.9)	67 (23.4)	
None or primary (≤4th grade)	69 (4.9)	44 (5.0)	57 (13.8)	38 (20.2)		181 (7.0)	27 (9.4)	
Monthly household income (*n* = 2736)								
<500 EUR/person	585 (43.0)	530 (65.0)	300 (78.1)	148 (83.2)		1393 (56.3)	170 (64.6)	
500–1000 EUR/person	649 (47.8)	252 (30.9)	70 (18.2)	28 (15.7)	<0.001	917 (37.1)	82 (31.2)	0.03
>1000 EUR/person	125 (9.2)	33 (4.1)	14 (3.7)	2 (1.1)		163 (6.6)	11 (4.2)	
Individual health insurance plan (yes) (*n* = 2796)	629 (45.5)	217 (26.0)	60 (14.9)	19 (10.8)	<0.001	857 (34.0)	68 (25.0)	0.003
Obstetric characteristics								
Multiparous (*n* = 2763)	665 (48.7)	436 (52.6)	217 (56.4)	99 (53.8)	0.04	1270 (51.1)	147 (53.5)	0.45
Obstetric history (*n* = 2806)								
First pregnancy	700 (50.7)	393 (46.5)	168 (41.8)	85 (47.5)		1218 (48.2)	128 (46.2)	
Previous pregnancy(ies) with no complications ᵇ	463 (33.6)	314 (37.2)	171 (42.5)	74 (41.3)	0.01	916 (36.2)	106 (38.3)	0.78
Previous pregnancy(ies) with complications ᵇ	217 (15.7)	138 (16.3)	63 (15.7)	20 (11.2)		395 (15.6)	43 (15.5)	
Smoking during pregnancy (*n* = 2826)	205 (14.8)	61 (7.1)	17 (4.3)	8 (4.3)	<0.001	260 (10.2)	31 (11.1)	0.64
Complications during pregnancy ᶜ (*n* = 2825)	396 (28.6)	252 (29.5)	109 (27.3)	49 (26.3)	0.76	717 (28.1)	89 (32.1)	0.16
Twin pregnancy (*n* = 2890)	19 (1.3)	16 (1.8)	4 (1.0)	1 (0.5)	0.42	36 (1.4)	4 (1.4)	0.98
Delivery with any obstetric intervention ᵈ (*n* = 2784)	959 (70.2)	605 (72.0)	246 (62.4)	120 (65.2)	0.003	1754 (70.0)	176 (63.5)	0.03
Adverse neonatal outcome ᵉ (*n* = 2878)	208 (14.8)	150 (17.2)	49 (12.0)	25 (13.3)	0.08	384 (14.8)	48 (16.8)	0.36
Time elapsed since delivery (*n* = 2889)								
1–3 months	877 (62.0)	597 (68.4)	262 (63.6)	100 (52.9)		1669 (64.1)	167 (58.4)	
4–6 months	397 (28.0)	219 (25.1)	117 (28.4)	65 (34.4)	0.001	711 (27.3)	87 (30.4)	0.12
>6 months	141 (10.0)	57 (6.5)	33 (8.0)	24 (12.7)		223 (8.6)	32 (11.2)	

^a^ PALOP refers to Portuguese-speaking African countries. ᵇ Previous pregnancy complications were reported by the participants during the phone interview and included: anaemia, high blood pressure, preeclampsia, gestational diabetes, deep vein thrombosis, urinary tract infection, severe back pain, placenta praevia, placental abruption, preterm rupture of membranes, preterm labour, depression, and other rare complications. ᶜ Complications during pregnancy were retrieved from clinical records and included: high blood pressure, preeclampsia, gestational diabetes, acute pyelonephritis, placenta praevia, placental abruption, and other rare complications. ᵈ Obstetric interventions included instrumented delivery (vacuum or forceps), episiotomy, and caesarean section. ᵉ Adverse neonatal outcomes included preterm birth, low birth weight, congenital malformation, or admission to a neonatal intensive care unit.

**Table 2:** Associations between language proficiency and EPDS scores ≥10.

Proficiency in Portuguese		EDPS Score 0–9	EDPS Score ≥10				
		*n* (%)	*n* (%)	OR (95%CI)	*p*trend	aOR ^a^ (95%CI)	aOR ᵇ (95%CI)
Complete sample		*n* = 2604	*n* = 286	*n* = 2890		*n* = 2583	*n* = 2890
	Native	1314 (92.9)	101 (7.1)	1.00 (reference)	<0.001	1.00 (reference)	1.00 (reference)
	Full	775 (88.7)	99 (11.3)	1.66 (1.24–2.22)		1.71 (1.24–2.35)	1.63 (1.21–2.19)
	Intermediate	360 (87.4)	52 (12.6)	1.88 (1.32–2.68)		1.79 (1.22–2.65)	1.68 (1.16–2.42)
	Limited	155 (82.0)	34 (18.0)	2.85 (1.87–4.36)		2.64 (1.64–4.24)	2.55 (1.64–3.99)
Restricted to natives and recent migrants ᶜ		*n* = 1788	*n* = 191	*n* = 1979		*n* = 1780	*n* = 1979
	Native	1314 (92.9)	101 (7.1)	1.00 (reference)	<0.001	1.00 (reference)	1.00 (reference)
	Full	214 (84.3)	40 (15.7)	2.43 (1.64–3.60)		2.50 (1.60–3.92)	2.44 (1.61–3.69)
	Intermediate	143 (87.2)	21 (12.8)	1.91 (1.16–3.15)		1.89 (1.10–3.24)	1.69 (1.01–2.85)
	Limited	117 (80.1)	29 (19.9)	3.22 (2.05–5.08)		3.55 (2.09–6.02)	3.34 (2.03–5.49)

OR: crude odds ratios; aOR: adjusted odds ratios. ^a^ Models adjusted for maternal age, highest educational degree attained, marital status, having an individual health insurance plan, parity, delivery with intervention, and adverse neonatal outcomes, complete-cases analysis. ᵇ Adjusted models after multiple imputation. ᶜ Recent migrants are those who have been living in Portugal for ≤5 years.

## Data Availability

The data presented in this study are available on request from the corresponding author.

## Supplementary Materials

The following are available online at https://www.mdpi.com/article/10.3390/ijerph18094782/s1, Table S1: Maternal characteristics by interview status among women who consented to participate (*n* = 5272), Table S2: Associations between language proficiency and EPDS scores ≥11, and ≥13.
