# Supplementary material for: Language Proficiency and Migrant–Native Disparities in Postpartum Depressive Symptoms
Source: Int J Environ Res Public Health. 2021 Apr 29;18(9):4782. doi: 10.3390/ijerph18094782 (PMC8125143; doi:10.3390/ijerph18094782)
Supplement: Supplementary file 1 [file ijerph-18-04782-s001.zip › ijerph-1172438-supplementary.pdf]

Table S1. Maternal characteristics by interview status among women who consented to participate (n = 5272)

Characteristics	Not interviewed [a] (n = 2266)	Interviewed (n = 3006)	p
	n (%)	n (%)	
Sociodemographic characteristics			
Migrant (n = 5272)	1199 (52.9)	1573 (52.3)	0.67
Region of birth (n = 5272)			
Portugal	1067 (47.1)	1433 (47.7)	<0.001
Brazil	176 (7.8)	327 (10.9)	
PALOP [b]	697 (30.8)	713 (23.7)	
Other African countries	46 (2.0)	43 (1.4)	
Europe	180 (7.9)	347 (11.5)	
Asia	67 (3.0)	72 (2.4)	
America	33 (1.4)	71 (2.4)	
Age (years) (n = 5249)			
18-24	444 (19.8)	447 (14.9)	<0.001
25-34	1252 (55.8)	1669 (55.5)	
≥35	547 (24.4)	890 (29.6)	
Marital status (no partner) (n = 5238)	680 (30.5)	772 (25.7)	<0.001
Highest education level attained (n = 5059)			
Post-secondary (>12th grade)	537 (26.1)	1102 (36.7)	<0.001
Upper secondary (12th grade)	721 (35.1)	1088 (36.2)	
Lower secondary (9th grade)	521 (25.4)	605 (20.1)	
None or primary (≤4th grade)	275 (13.4)	210 (7.0)	
Obstetric characteristics			
Multiparous (n = 5006)	1217 (57.1)	1468 (51.1)	<0.001
Smoking during pregnancy (n = 5139)	320 (14.5)	300 (10.2)	<0.001
Complications during pregnancy [c] (n = 5146)	611 (27.7)	841 (28.6)	0.44
Twin pregnancy (n = 5272)	51 (2.2)	47 (1.6)	0.07
Delivery with any obstetric intervention [d] (n = 5065)	1407 (64.9)	2020 (69.7)	<0.001
Adverse neonatal outcomes [e] (n = 5241)	365 (16.2)	454 (15.2)	0.29

[a] Not interviewed women included those who were unreachable or did not complete the interview. [b] PALOP refers to Portuguese-speaking African countries. [c] Complications during pregnancy were retrieved from clinical records and included: high blood pressure, preeclampsia, gestational diabetes, acute pyelonephritis, placenta praevia, placental abruption, and other rare complications. [d] Obstetric interventions included instrumented delivery (vacuum or forceps), episiotomy, and caesarean section. [e] Adverse neonatal outcomes included preterm birth, low birth weight, congenital malformation, or admission to a neonatal intensive care unit.

Table S2. Associations between language proficiency and EPDS scores ≥11, and ≥13

Proficiency in Portuguese		EDPS score 0-10	EDPS score ≥11					EDPS score 0-12	EDPS score ≥13				
		n (%)	*n* (%)	OR (95%CI)	*p*trend	aOR[a] (95%CI)	aOR[b] (95%CI)	*n* (%)	*n* (%)	OR (95%CI)	*p*trend	aOR[a] (95%CI)	aOR[b] (95%CI)
Complete sample		*n* = 2676	*n* = 214	*n* = 2890		*n* = 2583	*n* = 2890	*n* = 2774	*n* = 116	*n* = 2890		*n* = 2583	*n* = 2890
	Native	1341 (94.8)	74 (5.2)	1.00 (reference)		1.00 (reference)	1.00 (reference)	1377 (97.3)	38 (2.7)	1.00 (reference)		1.00 (reference)	1.00 (reference)
	Full	792 (90.6)	82 (9.4)	1.88 (1.35-2.60)	<0.001	1.88 (1.32-2.69)	1.82 (1.30-2.54)	833 (95.3)	41 (4.7)	1.78 (1.14-2.80)	<0.001	1.91 (1.17-3.13)	1.73 (1.09-2.75)
	Intermediate	379 (92.0)	33 (8.0)	1.58 (1.03-2.42)		1.44 (0.89-2.30)	1.38 (0.89-2.15)	390 (94.7)	22 (5.3)	2.04 (1.19-3.50)		1.71 (0.93-3.14)	1.67 (0.96-2.92)
	Limited	164 (86.8)	25 (13.2)	2.76 (1.71-4.47)		2.43 (1.40-4.20)	2.47 (1.49-4.10)	174 (92.1)	15 (7.9)	3.12 (1.68-5.80)		2.14 (1.02-4.49)	2.51 (1.31-4.83)
Restricted to natives and recent migrants [c]		*n* = 1835	*n* = 144	*n* = 1979		*n* = 1780	*n* = 1979	*n* = 1902	*n* =77	*n* = 1979		*n* = 1780	*n* = 1979
	Native	1341 (94.8)	74 (5.2)	1.00 (reference)		1.00 (reference)	1.00 (reference)	1377 (97.3)	38 (2.7)	1.00 (reference)		1.00 (reference)	1.00 (reference)
	Full	219 (86.2)	35 (13.8)	2.90 (1.89-4.44)	<0.001	2.96 (1.82-4.82)	2.83 (1.80-4.45)	235 (92.5)	19 (7.5)	2.93 (1.66-5.17)	<0.001	3.57 (1.86-6.86)	3.27 (1.79-5.98)
	Intermediate	151 (92.1)	13 (7.9)	1.56 (0.85-2.88)		1.47 (0.76-2.86)	1.33 (0.71-2.52)	157 (95.7)	7 (4.3)	1.62 (0.71-3.68)		1.50 (0.60-3.77)	1.43 (0.61-3.35)
	Limited	124 (84.9)	22 (15.1)	3.22 (1.93-5.36)		3.28 (1.79-5.99)	3.19 (1.82-5.59)	133 (91.1)	13 (8.8)	3.54 (1.84-6.81)		3.18 (1.41-7.18)	3.43 (1.65-7.10)

OR: crude odds ratios; aOR: adjusted odds ratios. [a] Models adjusted for maternal age, highest educational degree attained, marital status, having an individual health insurance plan, parity, delivery with intervention, and adverse neonatal outcomes, complete-cases analysis. [b] Adjusted models after multiple imputation. [c] Recent migrants are those who have been living in Portugal for ≤ 5 years.
